# Economic evaluation of rituximab plus cyclophosphamide, vincristine and prednisolone for advanced follicular lymphoma

**DOI:** 10.1080/10428190701769665

**Published:** 2008-01-31

**Authors:** John Hornberger, Carolina Reyes, Deborah Lubeck, Nancy Valente

**Affiliations:** ^1^Cedar Associates LLC, Menlo Park, CA, USA; ^2^Department of Medicine, Stanford University, Stanford, CA, USA; ^3^Genentech Inc., South San Francisco, CA, USA; ^4^ICON Clinical Research, San Francisco, CA, USA

**Keywords:** Lymphoma, follicular lymphoma, indolent lymphoma, low-grade lymphoma, economics, costs, cost-benefit, rituximab, CVP

## Abstract

The addition of rituximab to cyclophosphamide, vincristine and prednisolone (CVP) for advanced follicular lymphoma increases median time to progression by 17 months. A US societal cost-effectiveness of R-CVP versus CVP is estimated for a representative 50-year-old patient. Progression-free survival (PFS) and overall survival are based on a randomized Phase III trial. Costs are estimated using Medicare reimbursement rates and published drug price data, and include drug and administration costs, adverse events, treatment of relapses, and end-of-life care. Utility estimates are derived from the literature and a 3% discount rate is employed. Mean overall survival is projected to be 1.51 years longer for patients assigned to R-CVP versus CVP. The cost per quality-adjusted year of life gained is $28,565. The utility associated with stable or progressive disease and the unit drug cost of rituximab most influence the findings. The cost-effectiveness ratio of R-CVP compared with CVP is projected to be cost-effective in the United States under a range of sensitivity analyses.

## Introduction

Approximately 22% of the more than 55,000 patients diagnosed this year in the United States with non- Hodgkin lymphoma (NHL) are classified as follicular [1]. The disease course of follicular lymphomas isconsidered slowly progressive, involving repeatedrelapses and a median survival of 6–11 yearsdepending on the stage of disease [1–6].

Initial treatment of follicular lymphoma withchemotherapy can often achieve a response, butalmost all patients relapse within 4–5 years. Although there is no consensus on first-line therapyof follicular lymphoma, single agents such aschlorambucil or combination regimens such ascyclophosphamide, vincristine and prednisolone(CVP) or cyclophosphamide, doxorubicin, vincristineand prednisolone (CHOP) are commonly usedtreatment regimens.

Rituximab (Rituxan®, Genentech, Inc., South SanFrancisco, CA) is a genetically engineered chimericmurine/human monoclonal antibody directed againstthe CD20 antigen found on the surface of normaland malignant B lymphocytes. The antibody is anIgG1 kappa immunoglobulin containing murinelight- and heavy-chain variable region sequencesand human constant region sequences. Rituximabwas found to cause lysis of CD20+ lymphoma cellsvia complement-mediated cytotoxicity, antibodydependentcellular cytotoxicity and, directly, bycausing apoptosis. Rituximab has demonstratedsingle-agent activity in the treatment of patientswith relapsed or refractory low-grade or follicular,CD20+ B-cell NHL [7], which led to the initialapproval for this indication in 1997. In a Phase IIIclinical trial, Marcus et al. studied the addition ofrituximab to the widely used combination regimen of CVP [8]. The trial demonstrated that rituximab used in combination with CVP (R-CVP), compared with CVP, increased overall and complete rates response(overall: 81% versus 41%; complete: 57% versus10%; p50.001) [8]. Importantly, R-CVP alsosignificantly prolongs median time to progressionfrom 15 to 32 months (p50.0001). This trialformed the basis for the FDA approval in September2006 of the expanded use of rituximab in combinationwith CVP for patients with previously untreated CD20+, B-cell, follicular NHL.

The objective of this study is to determine whetherR-CVP is a cost-effective alternative to CVP for firstlinetreatment of advanced follicular lymphoma. Thefactors that influence the cost-effectiveness of R-CVPalso are examined.

## Materials and methods

### Analytical framework

The principles of decision-theoretical modelingcommonly applied in health economic appraisalsare used in this analysis. The model framework isbased on the Markov model, which provides aconvenient way of modeling disease progressionthat monitors events occurring in a hypotheticalcohort of patients under various scenarios. Keyparameters of the model are based on balancedsummary of clinical evidence and reasonable assumptions.In a Markov model, the patient may be inone of a finite number of states of health and eventsof interest are modeled as transitions from one stateto another. For each state, analysts assign a utilityused as an adjustment factor for quality of life. Utilityweights typically range from 0 to 1, where 0represents death, 1 represents perfect health; thevalues between 0 and 1 represent degrees betweenthese extremes. The contribution to total utility,commonly referred to as quality-adjusted life years(QALYs), of a particular state depends on the lengthof time spent in a state multiplied by the utility of thatstate. The model includes 3 states: (1) time untilprogression or death, referred to as progression-freesurvival (PFS), (2) time after progression and (3)death.

### Target population

The model includes the costs and effects of R-CVPtreatment compared with CVP in a representative patient with advanced follicular lymphoma. The target population consists of patients age 18 yearsand older with Ann Arbor Stage III or IV follicular NHL with International Working Formulation(IWF) categories B, C, or D (WHO follicular grades 1–3), who have Eastern Cooperative Oncology Group (ECOG) performance score between 0 and 2, and have untreated and measurable disease.

Among all enrolled patients in the pivotal trial,median age was 53 years, 70% had Ann Arbor StageIV disease, 4.4% had hemoglob in level 5100 g/L,26% had serum LDH4 upper limit of normal, 64% had bone marrow involvement and 32% had 43 nodal sites with diameters greater than 3 cm. The Follicular Lymphoma International Prognostic Index (FLIPI) score was greater than 2 in 53% of patients [8].

### Interventions

**Figure 1:**
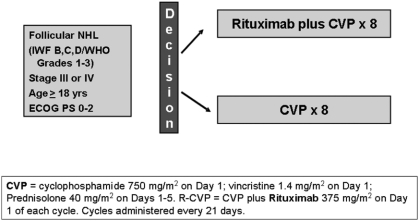
First-line R-CVP First R versus CVP.

A cycle of CVP consists of cyclophosphamide 750 mg/m^2^ IV Day 1, vincristine 1.4 mg/m^2^ up to 2 mg/m^2^ IV Day 1, and prednisolone 40 mg/m^2^ orally Days 1–5 of each cycle. R-CVP consists of CVP plus rituximab 375 mg/m^2^ given on the first day of each cycle. CVP +/7 R cycles are repeated every 21 days for up to 8 total cycles (Figure 1). Patients who did not achieve a partial response after 4 cycles of therapy were removed from the trial. If a rituximab-induced infusion reaction occurs, therapy is interrupted and all symptoms must resolve before rituximab is continued, or CVP restarted. Dosages of cytotoxic drugs were reduced if grades 2/4 neurological or grade 3/4 hematological toxicity occurred.

### Progression-free survival and overall survival

**Figure 2:**
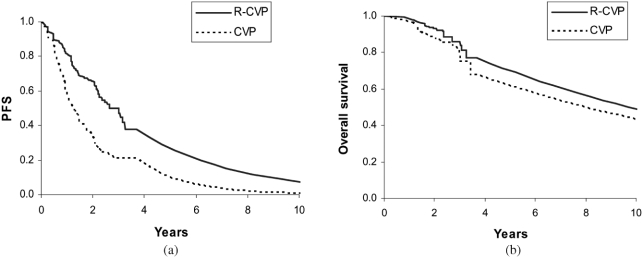
(a) Observed survival based on the trial data and then predicted using hazard ratios. (b) Observed progression-free survival and then predicted using hazard ratios.

Progression-free survival (PFS) and overall survivalfor the first 4 years after initiating treatment are basedon Kaplan–Meier survival analyses of a pivotalclinical trial (Figure 2) [8]. PFS and overall survivalare extrapolated beyond the trial's 4 years of followupbased on published findings of long-term observational studies. For example, Solal-Celigny et al.reported the prognosis of 4167 patients with follicular lymphoma diagnosed between 1985 and 1992 [5]. Applying an annual mortality risk of 6.9% approximately replicates the overall survival reportedin this and related studies [3–6].

### Costs

The costs in these models are obtained from the initial regimen of chemotherapy administered to all the patients. Unit drug costs are derived from Medicare J-codes (CMS-approved reimbursement rates) [9]. The CVP regimen requires 5 doses of 40 mg/m^2^ of prednisolone per cycle. For the average adult, this requires a net dose of 1.72 m^2^6 100 mg/m^2^65 ¼ 860 mg. At a cost of less than $0.01/mg, the cost of prednisolone in one cycle of CVP is $5. Table I shows the calculation of the costs of CVP. The actual dose given is calculated from the normalized dose and a standard BSA (1.72 m^2^). The recommended does is calculated from the product insert recommendations. The actual dose divided by the recommended dose equals the percent of the recommended administered. The cost per cycle is the cost of one cycle of the recommended dose, calculated using Mosby 2006 drug costs. Finally, the cost per course equals the product of the cost per cycle, the number of cycles per course and the percent administered. The same cost for CVP is used in both arms. Rituximab is sold as 10 mL (100 mg) and 50 mL (500 mg) vials, with equal unit cost regardless of vial size ($5.28/mg). Rituximab is assumed to be purchased in 100 mg increments and excess drug per vial would be wasted. The dose is multiplied by the cost per mg to give the cost per cycle. The cost per course equals the product of this cost per mg and the number of cycles per course (Table II).

**TABLE I.** Calculation of costs of CVP.

	CVP			R-CVP		
		
	CTX	Vincristine	Prednisolone	CTX	Vincristine	Prednisolone
Normalized dose given (divided by BSA)	4866	7.1	1323	5315	7.7	1471
Recommended dose	10,320	19.3	6880	10,320	19.3	6880
Actual dose given	8370	12.2	2276	9142	13.2	2530
Percent	81.1%	63.4%	82.7%	88.6%	68.8%	36.8%
Cost per cycle	$47.40	$13.37	$13.32	$46.60	$13.37	$13.32
Cost per course	$302.35	$67.81	$88.11	$330.25	$67.81	$88.11
			$458.27			$501.76

CTX, cyclophosphamide; BSA, body surface area.

**TABLE II.** Rituximab cost calculation.

Dosing information	Estimate
Intended number of cycles	8
Mean body surface area (m^2^)	1.72
Prescribed dose (mg per m^2^)	375
Dose per cycle (mg)	645
Dose purchased (mg)*	700
Cost per cycle	$3372
Percent of dose given	89.1%
Cost per course	$24,034

* One 500 mg vial (50 mL) and two 50 mg vials (10 mL).

Administration costs are calculated by multiplying the required number of hours of infusions by the cost per hour listed for the appropriate current procedural terminology (CPT) codes [9]. The number of hours required to receive rituximab are based on the administration section and toxicity profile of rituximab monotherapy reported in the product insert. For the R-CVP arm, both rituximab and CVP are administered together, whenever possible, to minimize costs. Rituximab takes the longest time to infuse and thus drives the administration costs. Based on data from the product insert and a clinical expert (NV), the average time required for the first administration is assumed to be 5.4 h, and 4.4 h for subsequent administration. This takes into account the estimate from clinical experts regarding the proportion of patients who develop mild and severe infusion reaction (approximately 77% of patients in the first administration experience an infusion reaction, requiring the slowing of administration). Table III shows the cost calculation for the R-CVP arm for the first cycle, and subsequent cycles. The CVP arm is simpler requiring only 2 costs: the initial administration plus one additional infusion (prednisolone is administered orally and thus does not incur administration costs).

**TABLE III.** R-CVP arm administration costs.

Administration costs	CPT Code	Fee	Amount
First cycle			
Initial administration cost	96413	$172.81	1
Additional hour cost	96415	$39.03	4.4
Additional infusion cost	96417	$84.51	2
Total Cost		$513.56	
Subsequent			
Initial administration cost	96413	$172.81	1
Additional hour cost	96415	$39.03	3.4
Additional infusion cost	96417	$84.51	2
Total Cost		$474.53	

The known safety profile of rituximab monotherapy includes infusion reactions consisting of fever, chills/rigors, nausea, angioedema, asthenia and headache. Based on the product insert, symptoms were found to be most common with the first rituximab monotherapy infusion (77%), decreasing in incidence to 30% with the fourth infusion, and 14% with the eighth infusion [10]. An infusion reaction is assumed to prolong administration times, thereby increasing the costs of administration. Only Grade 3 and 4 adverse events with at least 2% rate difference between the two arms of the trial are considered as contributing substantially to medical costs. The costs of treatment of adverse events are multiplied by the probability of occurrence reported in the trial. Both fatigue and granulocytopenia occur in higher frequencies in the R-CVP arm. Two options were available for the treatment of granulocytopenia, filgastrim, or pegfilgastrim. Filgastrim results in higher costs; hence, it is used so as to bias the analysis against R-CVP. The costs of fatigue are estimated from the cost of an office visit that would result from the condition.

The cost of subsequent treatment regimen is calculated from the average price of the most common regimens recommended by the National Comprehensive Cancer Network (NCCN) for NHL (aside from those including rituximab) [11]. Rituximab monotherapy is also indicated for relapsed/ refractory follicular lymphoma. The following treatment regimens are considered: chlorambucil, cyclophosphamide, CHOP, fludarabine, FMD (fludarabine, mitoxantrone, dexamethasone), rituximab monotherapy, R-CHOP, R-fludarabine and R-FMD. Moreover, based on preliminary trial evidence [12–14], some physicians are offering rituximab after initial therapy in patients who have achieved a complete or partial response. We therefore include costs associated with such regimens in the model, assuming that 70% of patients would receive at least one additional course of rituximab (7 cycles on average).

The subsequent treatment would have no additional effect on OS on either arm and would add a measure of disutility as well to the six-month cycle in which it was applied. The model applies one round of subsequent treatment at the median time to progression and one year thereafter for each arm. Based on NCCN guidelines, salvage regimens are assumed to be available to relapsed patients and that these regimens were tried in equal proportions. The average costs for drugs and administration are included to account for one round of salvage therapy. Table IV shows the six different regimens, their cost and the average cost of salvage therapy.

**TABLE IV.** Salvage therapy regimens and costs over 6 months.

Regimen	Utilization (%)	Cost
Chlorambucil	6	$935
Cyclophosphamide	6	$1541
CHOP	6	$3829
Fludarabine	6	$9001
FMD	6	$22,084
Rituximab-containing regimens*	70	$29,084
Average		$23,206

* Includes R-CHOP, R-CVP, R-COP, R-F, R-FMD, with patients receiving at seven cycles of rituximab.

The prospect that some patients may undergo stem-cell transplantation (SCT) is included in the model. A comprehensive search of PubMED failed to identify relevant papers regarding the proportion of patients with advanced follicular NHL who undergo SCT as part of their subsequent therapy. Based on a limited survey of NHL experts' opinions, an estimate of 10% was used for patients that undergo SCT based on those who are alive by year 7. The cost of this procedure is derived from a review of costs [15] and the average of the costs of SCT procedures performed for NHL patients was selected. The1992 cost was updated by applying an inflation factor to update the mean cost to 2006 dollars Costs of end-of-life care also are included in the analysis. Hoover et al. stated that the costs of health care increase significantly in the last year of life [16]. More specifically, they calculate the cost of the terminal year of life, based on data from the Medicare Current Beneficiary Survey.

The analysis includes the costs of adverse events. Only Grade 3 and 4 events are considered, and only if the difference in prevalence between the two arms reported in the trial exceeded 2%. The costs are calculated by multiplying the incidence by the unit costs of treatment.

### Utilities

Cost-effectiveness models calculate the incremental cost of a given technology per benefit gained. Though many measures of benefits exist, the QALY has been widely adopted as a standard measure in cost-effectiveness research. Because chemotherapy causes a significant decrease in quality of life, its positive effects (such as gain in overall survival) are partially negated by its toll on quality of life at the time of administration.

The model incorporates the effects of QOL in different scenarios by assigning utility weights for follicular lymphoma [17] and “disutility” tariffs to certain scenarios, such as chemotherapy, SCT and end-of-life care [18]. This accounts for a day with chemotherapy holding less value than a day in perfect health. Through sensitivity analyses, a wide range of utility values are explored to determine their effect on the outputs of the model.

### Other assumptions

To account for the changing value of money over time, two time-discount parameters are included in the model. The first is the societal time-discount rate, a correction for costs and benefits incurred at future dates. Because a dollar or benefit incurred today typically is considered preferable to a dollar or benefit incurred later, the model applies a standard time-discount rate to all costs and benefits incurred in future years [19]. The medical consumer price index is a parameter published by the Bureau of Labor Statistics, which adapts prices to reflect currents trends in the rising prices of healthcare [20]. This term inflates health care costs incurred in future years.

The time horizon is set to 30 years, which in this population, approximates a lifetime model. The ramifications of choosing different time horizons are explored. Table V summarizes all the variables discussed and the evidence sources.

**TABLE V.** Summary of base-case estimates, ranges for sensitivity analyses and quality of the evidence.

	Base-case estimate			
			
Parameter	R-CVP	CVP	Range for sensitivity analyses	Quality of evidence
Utilities				
Follicular lymphoma pre-progression	0.805		0.70 to 90	B
Post-progression	0.618		0.52 to 0.72	B
Tariffs				
Chemotherapy	70.15		70.3 to 0	C
Stem cell transplantation	70.20		70.4 to 0	C
End-of life (last 6 months)	70.30		70.6 to 0	C
Costs				
Chemotherapy drugs				
Rituximab	$24,034	−	±25%	A
CVP	$502	$458	±25%	A
Chemotherapy administration	$3,529	$1,702	±25%	A
Adverse events	$580	$95	±25%	A
After treatment				
Follow-up tests and visits	Approximately $142 every 3 months		±25%	B
Follow-up treatment Stem cell transplantation	$23,206		−25% to +100%	B
Stem cell transplantation	$75,352		±25%	B
End-of-life care	$21,463		±25%	B
Other variables				
Societal time discount rate	3%		0% − 5%	A
Time horizon	Lifetime		5 years to lifetime	B

CVP, chemotherapy regimen containing cyclophosphamide; vincristine and prednisolone.

**Figure 3:**
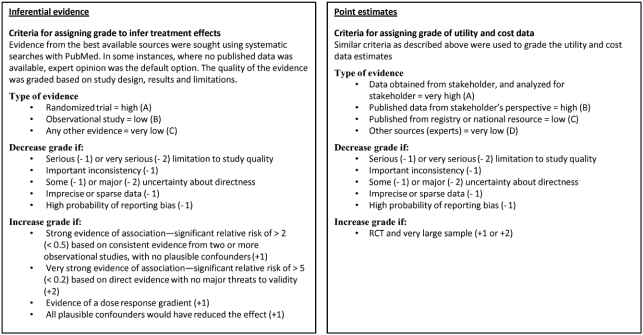
Quality of the evidence − grading system.

See Figure 3 for grading system.

Cost of laboratories per month; increase by $841 for periodic CT scans in first 2 years.

### Quality of evidence

Evidence was sought from the best available sources, including the randomized trial itself, based on systematic searches of the literature using PubMed and SciSearch. In instances where no published data was available, evidence was based on expert opinion. The quality of the evidence is graded based on study design, results and limitations, using two grading systems [21,22]. The first system assesses evidence pertaining to inference about treatment effects. The grading system assumes that findings from a well-controlled randomized clinical trial represent Level A evidence, whereas findings from an observational study represent level B evidence. Level C evidence derives from other sources, such as expert opinion or small case series. The grading level is altered by one or two levels based additional criteria, such as strength of association, consistency of findings, level of potential reporting bias, concerns about study limitations and generalizability of the findings.

For the second grading system, Level A evidence represents data obtained from the stakeholder. For example, if the analysis is done from the perspective of the Centers for Medicare and Medicaid services (CMS), Level A evidence would be based on CMS claims analyses. Another example is obtaining evidence from an utility assessment project where the participants are a random sample from the stakeholder's stated constituency. Level B evidence involves obtaining estimates that pertain to the stakeholder perspective, but was not directly analyzed for this project (e.g., from a review of the literature). Level C evidence represents data obtained from other database sources, such as utility or cost registries. Level D evidence represents data from other sources, such as Delphi panel of experts. The grading level can be altered to reflect strengths and limitations of the study. It is worth noting that utility or cost data from a randomized control trial may be graded from A to C, depending on the particular relevance of the information to the stakeholder. For example, cost data analyzed in a trial in which most of the participants were from a country or healthcare system substantially different from that of the stakeholder has lower relevance, and so may be assigned to Level C evidence. Figure 3 shows the criteria used to grade the data on treatment effects and the cost and utility data. Table V shows the grades for all the data and their sources that are used in the present analyses.

## Results

R-CVP is projected to increase mean PFS by 1.93 years compared with CVP alone. R-CVP increases mean overall survival by 1.51 years and QALYs by 0.93 years (Table VI).

**TABLE VI.** Base-case results.

Endpoint	R-CVP	CVP	Difference
OS (yr)	13.68	12.17	1.51
PFS (yr)	3.78	1.84	1.93
QALY			
Remission	6.40	5.51	0.89
Chemotherapy	70.15	70.15	0.00
Salvage treatment	70.19	70.22	0.03
End of life	70.21	70.22	0.01
Total	5.85	4.93	0.93
Costs			
Chemotherapy drugs	$24,536	$458	$24,078
Chemotherapy administration	$3529	$1702	$1827
Adverse events	$580	$95	$485
Follow-up tests and visits	$13,348	$12,051	$1297
Stem cell transplantation	$5123	$4514	$609
Salvage treatment	$34,466	$36,610	−$2144
End of life	$24,025	$23,737	$287
Total	$105,607	$79,168	$26,439
Cost per life-year gained			$17,504
Cost per QALY gained			$28,565

OS, overall survival; PFS, progression-free survival; QALY, quality-adjusted life years; CVP, chemotherapy regimen containing cyclophosphamide, vincristine and prednisolone.

Drugs, administration and follow-up tests and visits incur the highest added costs associated with adding rituximab to CVP. The total cost difference between the two arms for the trial was $26,439. Treatment with rituximab incurs additional costs in all categories, except for salvage treatment.

The cost of rituximab alone comprises 92% of the total cost difference between the regimens. All other cost categories had far less impact on total cost and this is confirmed in sensitivity analyses. It is important to note that while salvage therapy, endof-life care and post-treatment follow up incur costs above $20,000, the difference in these costs between the two treatment arms is minimal and thus does not significantly affect the results.

Administration costs varied between R-CVP and CVP because of the longer time required to administer rituximab. The addition of rituximab to the CVP regimen results in a higher incidence of adverse events, thereby increasing those costs. Higher follow up costs were mainly due to years of life gained, resulting in more follow-up care. Increased survival also increases the costs due to SCT because more patients are alive after 7 years to undergo the procedure. The trial data showed a cost-effectiveness ratio of $28,565 per quality-adjusted life-year gained.

### Sensitivity analyses

**Figure 4:**
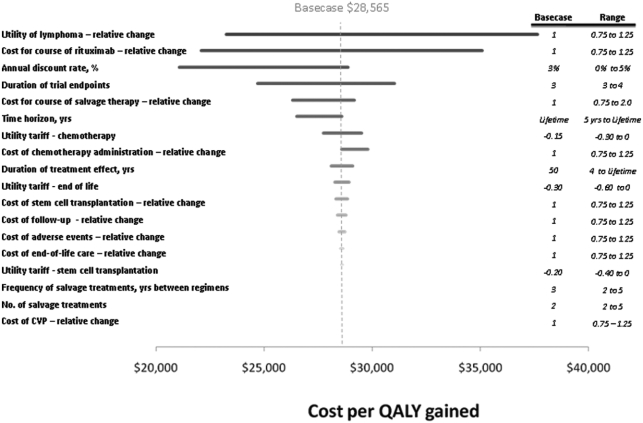
Figure 4. Sensitivity analyses.

Figure 4 shows the effects of changing the value of the inputs on the outcome of the model. The utility of follicular lymphoma and the cost for a course of rituximab are shown to most influence the cost per QALY gained. Lower utility associated with follicular lymphoma is associated with higher cost-effectiveness ratio. The cost of rituximab also contributes significantly to the model outcomes. Changing the cost for a course of rituximab by +25% varies the cost-effectiveness ratio by $12,983. In none of the sensitivity analyses did the cost-effectiveness of R-CVP versus CVP exceed $50,000 per QALY gained.

## Discussion

Therapeutic options for patients with follicular lymphoma are extensive, ranging from single-agent chemotherapy to combination chemotherapy regimens. Patients typically achieve high complete response rates (*60%) with frontline therapy, with remission durations of up to 3 years [3,23]. Eventually, patients experience relapse and are treated with a series of chemotherapeutic regimens over their lifetime, with diminishing efficacy.

The development of rituximab led to a clinical trial to assess the clinical outcome and patient benefit of the addition of rituximab to CVP chemotherapy in frontline treatment [8]. The trial showed that rituximab added to a CVP regimen significantly increased PFS. Although overall survival was not a primary endpoint of the trial (that is, the trial was not powered to show statistical significance by 4 years), the difference in survival in the two arms was 8% apart at 4 years, showing no trend towards convergence thereafter. R-CVP, therefore, is projected to increase overall lifetime costs for frontline treatment of advanced follicular lymphoma; however, the cost-effectiveness ratio of R-CVP versus CVP is less than $30,000 per QALY gained.

Sensitivity analyses show that the cost for adding rituximab to CVP and the utility associated with follicular lymphoma were the variables that most influenced cost-effectiveness. Because both treatment arms incur the CVP drug costs, increasing or decreasing these costs has minimal effect on the cost-effectiveness. It is important to note that many of the costly categories (e.g., salvage therapy) have little effect in this model because over a patient's lifetime with follicular lymphoma, few patients will incur these costs. The influential variables, therefore, are not necessarily the absolute costs of each procedure, but the total difference in costs between each arm. The direct costs of the two arms differ mainly because of the additional cost of rituximab. The benefits gained from the added drug stem from increased efficiency in the form of delayed relapse.

Other regimens are also used for first-line treatment of follicular NHL. In this study, we focused on the cost-effectiveness of rituximab used in combination with CVP. Our approach, however, could provide a basic framework for cost-effectiveness analyses of other induction regimens where evidence of patient benefit is demonstrated in randomized Phase III studies, such as trials of R-CHOP versus CHOP [24], R-CHVP versus CHVP [25], R-MCP versus MCP [26] and R-FCM versus FCM [12,27]. The sensitivity analyses show that the cost of Rituximab is among the most important factors influencing cost-effectiveness, especially compared with the other components of the chemotherapy regimen. All regimens mentioned above use less or the same number of cycles as used in the current study; hence, the additional costs of chemotherapy is unlikely to be higher than reported here. Additionally, Schulz et al. [28] reported a detailed metaanalysis of rituximab combination therapies, showing lower hazard ratios (0.42–0.60) for overall survival than reported by Marcus et al. (0.70) [28] A formal analysis of these other regimens therefore would likely demonstrate cost-effectiveness ratios that are even lower than what we found with R-CVP compared with CVP.

The cost-effectiveness of rituximab for first-line follicular lymphoma has yet to be formally assessed in other countries. The sensitivity analyses show that cost of Rituximab is one of the key influencers of the cost-effectiveness ratio. In many countries, rituximab is reimbursed at a lower level–adjusted for currency exchange rates–than in the United States. Hence, rituximab may be found to be more cost-effective outside of the United States. For other indications, such as diffuse large B-cell lymphoma, European health technology groups have concluded that a rituximab-based regimen, R-CHOP, is cost-effective and, as such, rituximab has been reimbursed for this indication. The extent to which health technology groups conclude that rituximab provides sufficient value for money for first-line follicular lymphoma also depends on the stakeholder's willingness to reimburse for health technologies; in other words, the country's threshold of acceptable cost-effectiveness.

These analyses should be interpreted in light of the study's potential limitations. First, clinical trial data was only available up to 4 years, whereas patients with follicular lymphoma are reported to have a median survival of 6 to 11 years [1–6]. To capture the implications of frontline chemotherapy with or without rituximab, survival is estimated after 4 years based on hazard ratios reported in the literature. Experience in other economic analyses of oncology interventions reveals that some clinicians are ambivalent about using non-trial data to estimate treatment effects beyond the duration of the trial. However, technology assessment guidelines (e.g., ISPOR or US Public Health) recommend estimating the potential treatment effect over the potential duration of the illness. To reconcile these different perspectives, the time horizon was selected that encompasses the patient's entire lifetime, but the duration of treatment effect was varied from 5 years to a lifetime. Even when setting the treatment effect to only 5 years, the cost-effectiveness ratio increases only slightly from $28,565 to $34,128. The extrapolations have minimal effect on the cost-effectiveness of R-CVP.

Besides the duration of treatment effect, the second most influential variable is the cost of rituximab. The cost-effectiveness ratio is composed of two parts: the additional price of the new technology in the numerator and its added benefit in the denominator. In practice, the cost of a technology and its effect on overall survival can often approximate the outcome of the all-inclusive model. Therefore, it is unsurprising that altering the cost would have a large effect on the results. The cost of rituximab alone does in fact influence the cost-effectiveness.

Based on published BLS data, the rate of medical inflation is higher than the discount rate [20]. This has the effect of making future costs more expensive than present costs. As with all the inputs, the ramifications of this assumption were explored with sensitivity analyses. The societal time discount rate is among the top five sensitive inputs in the model. A discount rate of 3% has been widely accepted [19], and choosing a value less than the medical inflation rate biases the model against R-CVP, because the regimen causes other costs to be delayed, which in turn increases them as result of the inflation factor.

The present analysis is consistent with previously reported cost-effectiveness analyses of rituximab for other NHL indications. In diffuse large B-cell lymphoma, R-CHOP is cost-effective relative to CHOP at less than $20,000 per QALY gain [29–31].

Rituximab monotherapy also has been reported to be cost-effective compared with observation only from a Canadian healthcare perspective in the maintenance treatment of relapsed/refractory follicular lymphoma [32].

In summary, first-line treatment of R-CVP compared with CVP alone is likely to result in a cost effectiveness ratio of approximately $30,000 per QALY gained. Cost-effectiveness ratios less than $100,000 per QALY gained are typically considered affordable in the US oncology marketplace [33].
